# Cryo-EM structures of prion protein filaments from Gerstmann–Sträussler–Scheinker disease

**DOI:** 10.1007/s00401-022-02461-0

**Published:** 2022-07-12

**Authors:** Grace I. Hallinan, Kadir A. Ozcan, Md Rejaul Hoq, Laura Cracco, Frank S. Vago, Sakshibeedu R. Bharath, Daoyi Li, Max Jacobsen, Emma H. Doud, Amber L. Mosley, Anllely Fernandez, Holly J. Garringer, Wen Jiang, Bernardino Ghetti, Ruben Vidal

**Affiliations:** 1 Department of Pathology and Laboratory Medicine, Indiana University School of Medicine, 635 Barnhill Dr., Indianapolis, IN 46202 USA; 2 Department of Biological Sciences, Markey Center for Structural Biology, Purdue University, West Lafayette, IN 47906 USA; 3 Center for Proteome Analysis and Center for Computational Biology and Bioinformatics, Indiana University School of Medicine, Indianapolis, IN 46202 USA; 4 Department of Biochemistry and Molecular Biology, Indiana University School of Medicine, Indianapolis, IN 46202 USA; 5 Stark Neurosciences Research Institute, Indiana University School of Medicine, Indianapolis, IN 46202 USA

**Keywords:** GSS, APrP, Cryo-EM, Neurodegeneration

## Abstract

Prion protein (PrP) aggregation and formation of PrP amyloid (APrP) are central events in the pathogenesis of prion diseases. In the dominantly inherited prion protein amyloidosis known as Gerstmann–Sträussler–Scheinker (GSS) disease, plaques made of PrP amyloid are present throughout the brain. The c.593t > c mutation in the prion protein gene (*PRNP*) results in a phenylalanine to serine amino acid substitution at PrP residue 198 (F198S) and causes the most severe amyloidosis among GSS variants. It has been shown that neurodegeneration in this disease is associated with the presence of extracellular APrP plaques and neuronal intracytoplasmic Tau inclusions, that have been shown to contain paired helical filaments identical to those found in Alzheimer disease. Using cryogenic electron microscopy (cryo-EM), we determined for the first time the structures of filaments of human APrP, isolated post-mortem from the brain of two symptomatic *PRNP* F198S mutation carriers. We report that in GSS (F198S) APrP filaments are composed of dimeric, trimeric and tetrameric left-handed protofilaments with their protomers sharing a common protein fold. The protomers in the cross-β spines consist of 62 amino acids and span from glycine 80 to phenylalanine 141, adopting a previously unseen spiral fold with a thicker outer layer and a thinner inner layer. Each protomer comprises nine short β-strands, with the β1 and β8 strands, as well as the β4 and β9 strands, forming a steric zipper. The data obtained by cryo-EM provide insights into the structural complexity of the PrP filament in a dominantly inherited human PrP amyloidosis. The novel findings highlight the urgency of extending our knowledge of the filaments' structures that may underlie distinct clinical and pathologic phenotypes of human neurodegenerative diseases.

**Supplementary Information:**

The online version contains supplementary material available at 10.1007/s00401-022-02461-0.

## Introduction

Among hereditary neurodegenerative diseases, the dominantly inherited prion protein (PrP) cerebral amyloidoses include *Gerstmann–Sträussler–Scheinker disease* (GSS), *PrP cerebral amyloid angiopathy,* and *prion amyloidosis with variable phenotypes*; that are caused, respectively, by missense, nonsense, or insertional mutations in the prion protein gene (*PRNP)* [14]. GSS is characterized clinically by ataxia, pyramidal signs and dementia, and neuropathologically by the presence of extracellular deposits made of PrP amyloid (APrP) in the brain. Parenchymal APrP and spongiform changes of variable degree are the central neuropathologic alterations associated with a c.305c > t mutation in *PRNP*, resulting in a proline to leucine substitution at PrP residue 102 (P102L) in patients who belong to the pedigree originally reported by Gerstmann, Sträussler, and Scheinker [11, 17]. However, in several other genetic variants of dominantly inherited prion protein amyloidosis, intraneuronal Tau inclusions, identical to neurofibrillary tangles (NFTs) in Alzheimer disease (AD), are consistently found to coexist with APrP in the gray matter of the cerebral hemispheres and brainstem, but not in that of the cerebellum [12, 14, 18].

The c.593t > c mutation in *PRNP*, resulting in a phenylalanine (F) to serine (S) amino acid substitution at PrP residue 198 (F198S), was discovered in patients belonging to a large kindred from Indiana [6, 12, 21]. The *PRNP* mutant allele occurs in cis configuration to guanine at polymorphic coding base 385 (c.385g) of codon 129, resulting in a valine (V) variant at residue 129. Thus, the mutation carriers can be either homozygous (VV) or heterozygous methionine/valine (MV) at codon 129. This polymorphism appears to have a role in modifying the clinical phenotype of GSS (F198S) [6]. The *PRNP* (F198S) mutation carriers present clinically with a GSS syndrome characterized by cerebellar ataxia, dysarthria, and progressive pyramidal and parkinsonian signs. As the disease progresses, memory impairment and cognitive dysfunction become severe [8, 14]. Neuropathologically, the main finding is the presence of APrP deposits as multicentric plaques and diffuse deposits throughout the cerebral cortex, subcortical nuclei, cerebellum, and brainstem, with a distribution pattern different from that of amyloid β (Aβ) in AD [12–14]. Extracellular APrP deposits and intracytoplasmic Tau inclusions in the form of NFTs and neuropil threads are numerous in the advanced stages of disease; however, neuropathologic findings in non-symptomatic mutation carriers indicate that APrP deposition precedes the development of Tau pathology [14].

Recently, the biochemical properties and atomic structures of Tau filaments from GSS (F198S) have been characterized [18]. The filaments are made of three and four repeat (3R, 4R) Tau and the paired helical filaments (PHFs) are structurally identical to those extracted from the brains of AD patients [9, 18], an observation that suggests a common mechanism through which amyloids may trigger aggregation of Tau. It was from cases of GSS (F198S) that human APrP was isolated and characterized biochemically for the first time [40]; however, the atomic structure of the PrP filaments has not yet been determined. APrP deposits in GSS (F198S) are composed of N- and C-terminally truncated fragments of PrP, formed through proteolytic cleavage of the precursor protein [40]. It has been previously shown that the fragments vary in lengths and span approximately from residues G58 to E152 [4, 28, 40, 41]. The sequence of the APrP forming peptides does not include the F198S mutation, glycans or the glycosylphosphatidylinositol (GPI)-anchor [4, 5, 41]. Structural analysis of the cellular PrP (PrP^C^) protein has shown a largely disordered N-terminus and a folded C-terminal globular domain containing three α-helices and two short, antiparallel β-strands [32, 37, 49, 51, 53].

Numerous studies have analyzed recombinant PrP, synthetic PrP, PrP fragments, and scrapie PrP (PrP^Sc^) [16, 20, 22, 25, 26, 48, 49, 55], but the structural characterization of APrP from human brain tissues has proven difficult due to its insolubility and propensity to aggregate. Herein, we determine for the first time the high-resolution cryo-EM structures of human APrP isolated post-mortem from the brain of two GSS (F198S) patients. These structures may enable the generation of positron emission tomography (PET) tracers for PrP, and therapeutic interventions. This work, which represents a significant step towards the identification of the structures of the different amyloids causing neurodegeneration, may help in revealing the role of extracellular amyloid in the cascade of neurodegeneration.

## Materials and methods

### Neuropathology

The brains of the patients diagnosed with GSS (Supplementary materials; Figure S1) were harvested at Indiana University School of Medicine. The fresh brains were hemisected along the mid-sagittal plane. The right hemibrain was sliced coronally; the slices were frozen and stored at − 70 °C. The formalin-fixed left hemibrain of patient one has previously been used to investigate and describe the patient’s neuropathologic phenotype and to correlate the in vivo magnetic resonance and the Tau PET neuroimaging with the post-mortem anatomic and immunohistochemical findings [31]. The Thioflavin S method was used to visualize APrP plaques and NFTs. Neurodegenerative pathology was further analyzed using antibodies raised against PrP, 3F4 (1:800, Dr. Richard Kacsak, Staten-Island, New York, USA), and PrP 23-40, PrP 95-108, and PrP 220-231 (1:100, Dr. Piccardo and Dr. Ghetti), and against Tau, AT8 (1:300, Thermo Fisher Scientific). The signal from polyclonal or monoclonal antibodies was visualized using avidin–biotin, with either goat anti-rabbit immunoglobulin or goat anti-mouse as the secondary antibody followed by horseradish peroxidase-conjugated streptavidin and the chromogen diaminobenzidine. Immunohistochemical sections were counterstained with hematoxylin. Double immunohistochemical studies were performed using the Dako En-Vision Doublestain System (Dako) following the manufacturer’s instructions.

### Sanger DNA analysis

Genomic DNA was extracted from frozen brain tissue. Polymerase chain reaction (PCR) was performed for the amplification of the *PRNP* gene as described [18]. PCR products were sequenced on a CEQ 2000XL DNA analysis system (Beckman Coulter, Fullerton, CA).

### APrP extraction

APrP was extracted using a modified water extraction protocol [29]. Three grams of cerebellar gray matter of patients 1 and 2 were homogenized in 10 ml/g TE buffer (0.1 M Tris, pH 7.4, 5 mM EDTA), and protease inhibitors (Complete, 1 mM pepstatin, 100 mM TLCK-HCl, 200 mM TPCK, and 1 mM leupeptin; all from Roche Molecular). After homogenization, 2 mM CaCl_2_, 0.3 mg/ml collagenase III and 10 µg/ml DNase I (Thermo Fisher Scientific) were added. After 16 h at 37 °C, samples were centrifuged at 10,000 g for 30 min at 4 °C. The pellet was resuspended in 8 ml of 0.15 M NaCl and centrifuged at 10,000 g for 30 min at 4 °C. This step was repeated until the OD_280_ of the supernatant was less than 0.075. The resulting pellet was then resuspended in 10 ml/g dH_2_O and centrifuged at 30,000 g for 1 h at 4 °C. This step was repeated 5 times, and the final supernatant was centrifuged at 250,000 g for 2 h at 4 °C. The pellet was resuspended in 100 µl/g tissue in PBS with 0.5% sulfobetaine (SB3-14, Sigma), and stored at 4 °C.

### Western blotting

Samples were analyzed after methanol precipitation as previously described [3]. For Western blotting, samples were resolved on 15% Criterion Tris–HCl gel (BioRad) and transferred to polyvinylidene difluoride (PVDF) membranes (Sigma). Membranes were blocked in 5% milk in TBS plus 0.1% Tween 20. Primary antibody (3F4) was used at 1:750 dilution in blocking buffer.

### Negative stain electron microscopy (EM)

Negative stain EM was carried out as we previously described [18]. Briefly, a small droplet of resuspended amyloid pellet was placed on a carbon-coated copper grid (400 mesh, Ted Pella), allowed to evaporate partially, and filaments were negatively stained with Nanovan for 5 s at RT. Images were taken on a Tecnai G2 Spirit Twin scope equipped with an AMT CCD Camera.

### Cryo-EM and image processing

After sonication and addition of amphipol A8-35 (Thermo Scientific) to a final 0.02% concentration to disperse APrP fibrils, 3 µl of sample was applied on a glow-discharged graphene oxide coated 1.2/1.3 μm holey carbon-coated 300-mesh copper grids (Quantifoil) before vitrification using a Gatan CP3 cryo plunger in a BSL-2 biosafety hood. Cryo-EM movies were collected using Leginon [38] on a Thermo Fisher Scientific Titan Krios at 300 kV with a Gatan K3-direct electron detector in super resolution counting mode. The inelastically scattered electrons were removed using a Gatan quantum energy filter with a 20 eV slit width. Movies were gain and motion corrected, aligned, dose weighted, and then summed into individual micrographs using MotionCor2 [54] through Appion [23]. Contrast transfer functions (CTFs) for the motion corrected images were estimated using CTFFIND4 [33] through RELION 3.1 (Table S1) [34].

### Helical reconstruction

Helical reconstruction was carried out using RELION 3.1 software [19, 34]. Filaments were picked manually as end-to-end linear segments and extracted with a 1024 pixel box, down scaled to 256 pixels that cover the full cross over distance. The initial twist value was approximated from the observed crossover distance in raw micrographs and 2D class averages. Particles were forwarded to several rounds of 3D classifications using a featureless cylinder as the initial 3D model. Three different types of filaments were identified based on the distinct differences in the number of protofilaments from the 3D class averages. Multiple rounds of 3D classifications were performed to make the subsets more homogenous. Particles corresponding to the best subset of each filament type were then re-extracted with a variety of smaller boxes (240 and 320 pixels) without downscaling. Multiple rounds of 3D classification with a range of the regularization value *T* = 10–100 were performed to determine optimal 3D class averages. The resulting maps from the Refine3D module of RELION 3.1 were sharpened using phenix.auto_sharpen [43]. The FSC curves using half maps were calculated using trueFSC.py available in JSPR software [39].

### Model building

A central region of the 3D map of the filament was extracted using e2proc3d.py from the EMAN2 suite [42] for model building. The map region was also flipped along its *Z* axis to obtain a map with opposite handedness. Polyalanine models were built manually in COOT [7] in both flipped and unflipped maps in the forward and reverse directions, resulting in four polyalanine models. Phenix.sequence_from_map [44] was run with each of the maps and corresponding polyalanine models against all annotated sequences (8172 sequences) from human brain tissue. The LLG scores from phenix.sequence_from_map were plotted as a function of the sequence id and top-ranking sequence was identified as major prion protein (Uniprot ID: P04156), residues 80–141. The findMySequence method was then used to independently find the same prion protein that best explains the cryo-EM density map. The final model was refined to acceptable stereochemical parameters (Table S1) using the refinement suite of Phenix [1, 2]. A final round of refinement was carried out using Rosetta with helical symmetry constraints [50]. The cartoons illustrating amino acid packing in the XY plane perpendicular to the helical axis and the *Z*-position variations along the helical axis were prepared using ProCart (available at https://jiang.bio.purdue.edu/procart).

### Hypothetical packing model generation

Hypothetical packing models for Type IIa/b were generated using Chimera X version 1.3. First, the manual erasing tool was used to segment the density of one of the protofilaments of the high-resolution Type IIb map. For the hypothetical Type IIa model, the high-resolution Type IIb map was fit into the apparent pair of protofilament densities in the low-resolution Type IIa map. Then the single protofilament from the Type IIb map was fit into the third single density for the low-resolution Type IIa map. For the hypothetical Type IIb map, two copies of the high-resolution Type IIb map were used. First, the high-resolution Type IIb map was fit into the stronger density of the low-resolution Type IIb map. Then one copy was rotated 180° and fit into the weaker second pair of densities in the low-resolution Type IIb map. This positioning of the extra third or third and fourth filaments for the hypothetical models does not represent a fixed structure for the unresolved densities.

### Data availability

Cryo-EM maps have been deposited in the Electron Microscopy Data Bank (EMDB) under accession numbers EMD-26607 and EMD-26613 for Type I and Type IIb, respectively. Refined atomic models have been deposited in the Protein Data Bank (PDB) under accession numbers 7UMQ and 7UN5 for Type I and Type IIb, respectively. The mass spectrometry proteomics data generated in this study have been deposited to the ProteomeXchange Consortium [47] via the PRIDE partner repository with the dataset identifier PXD032402.

## Results

### Genetics

A single-nucleotide (thymine to cytosine) substitution at coding base 593 (c.593t >c) in codon 198 of one *PRNP* allele is present in the DNA of patients 1 and 2, resulting in a serine for phenylalanine amino acid change (F198S). Patient 1 is homozygous for an adenine to guanine substitution at coding base 385 (c.385a >g) that results in a valine variant at codon 129 (VV). Patient 2 is heterozygous at coding base 385 resulting in methionine and valine at codon 129 (MV).

### Amyloid deposits in GSS (F198S)

**Fig. 1:**
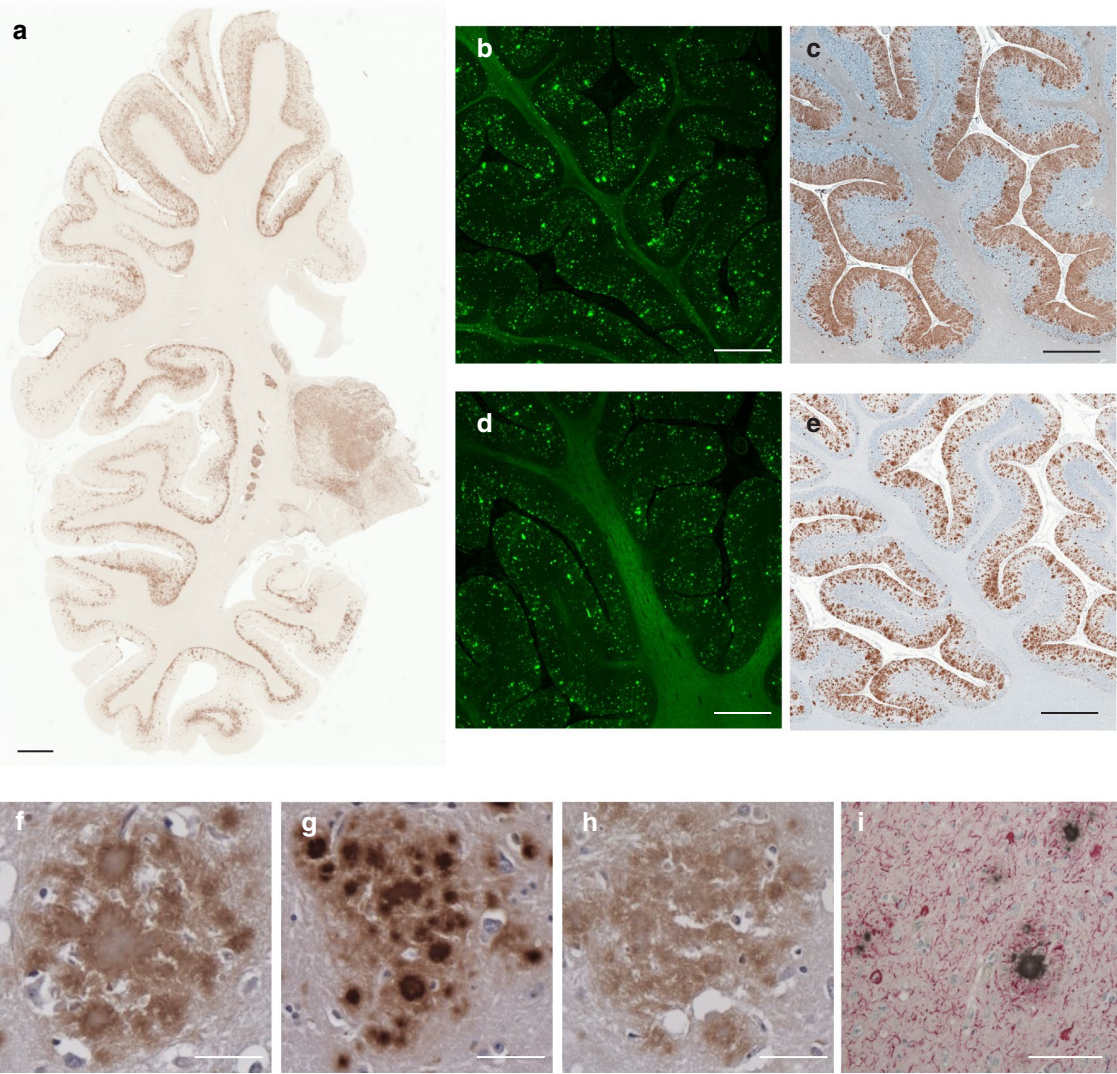
Histological and immunohistochemical features of GSS (F198S). Hemispheric coronal section shows the distribution of PrP deposits at the level of the cerebral cortex, hippocampus and thalamus in the GSS (F198S) patient 1 using antibody 3F4 (106-110) (**a**). Thioflavin S staining of a section of the cerebellum of patient 1 (**b**) and patient 2 (**d**). Immunohistochemical staining of a section of the cerebellum of patient 1 (**c**) and patient 2 (**e**) using antibody 3F4. Amyloid plaques in GSS (F198S) reactive for antibodies against 23-40 (f), 95-108 (**g**), and 220-231 (**h**) in the cerebral frontal cortex of patient 1. Double immunohistochemistry of Tau (AT8) and PrP (95–108) in the cerebral cortex in GSS (F198S) patient 1 (**i**). Scale bar, 4 mm (**a**), 2 mm (**b**–**e**), and 100 μm (**f**–**i**)

One of the hallmarks of the neuropathology of GSS (F198S) patients is the presence of numerous amyloid plaques (Fig. 1). APrP is mostly detected in gray structures of the cerebrum, cerebellum, and brainstem, in the form of amyloid plaques and diffuse deposits. APrP plaques may have a single or multiple cores (unicentric and multicentric plaques) and are predominantly found in layers 1, 4, 5 and 6 of frontal, insular, temporal, and parietal cortices (Fig. 1). Immunohistochemical analysis of brain sections shows that the amyloid cores are immunoreactive with antibodies binding to the central region of PrP (Fig. 1c, e, g) (Figure S2a), whereas those binding to the N- and C-termini label the periphery of the cores (Fig. 1f, h). In addition, Tau deposits, including NFTs and neuropil threads, coexist with APrP deposits in gray structures of the cerebrum and brainstem (Fig. 1i).

APrP was isolated from the cerebellum of patients 1 and 2 using a modified water extraction method. By negative staining EM, we observe the presence of fibrils and aggregated clusters (Figure S2b). Western blot analysis, carried out using antibody 3F4 (epitope located between aa 106-110), shows the presence of immunoreactive bands, including the previously reported bands at 8 and 11 kDa (Figure S2c) [4, 40].

Mass spectrometry analysis of the prion protein isolated from the cerebellum after trypsin digestion reveals the presence of peptides included in the PrP sequence from aa K23 up to R228, and thus the full protein without the N- and C-terminal signal peptides (Figure S3). The mass spectrometry analysis shows ragged N- and C-termini. A significant number of the detected peptides include the sequence starting near (or just before) amino acid G80 and ending between amino acids Y149 and S170. Numerous post-translational modifications were observed, including phosphorylation, acetylation, oxidation, and deamidation (Figure S3a, b). Analysis of the proteins present with the prion protein isolated from the cerebellum of patients 1 and 2 identified 2450 proteins in association with APrP deposits in patient 1 and 963 proteins in association with APrP deposits in patient 2. A total of 882 proteins were identified in both samples and constitute the APrP “interactome” (Figure S4), composed of proteins involved in the cytoskeleton, cytosol, focal adhesion membranes, extracellular exosomes, DNA replication, telomere organization, cytoplasmic translation, nucleosome assembly and RNA binding (Figure S4).

### APrP fibrils consist of two, three and four protofilament structures

**Fig. 2:**
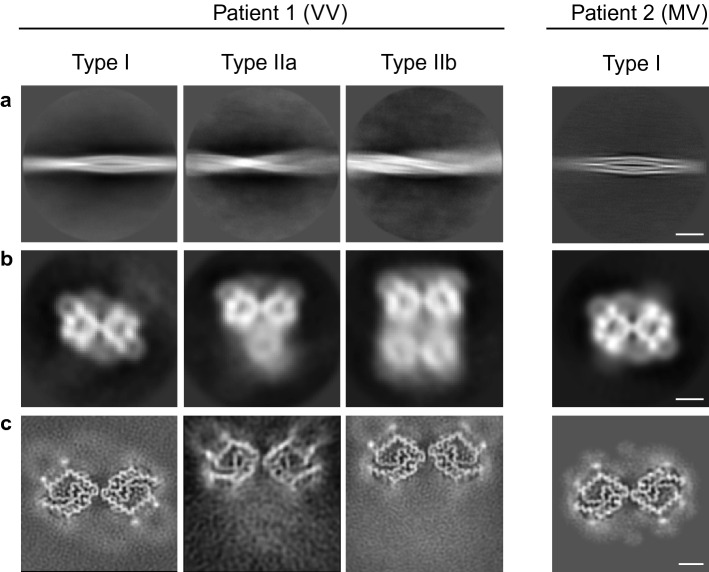
Cryo-EM reconstructions of APrP fibrils from GSS (F198S) patients 1 and 2. 2D classification of Type I and IIa/b filaments (**a**). Low-resolution initial cryo-EM maps, depicted as central slices (**b**). Final cryo-EM maps, depicted as central slices (**c**). Scale bars 200 Å (**a**), 25 Å (**b**), 50 Å (**c**)

APrP fibrils were dispersed using amphipol A8-35 for cryo-EM analysis (Figure S5a). Helical reconstruction of the prion filaments extracted from cryo-EM images reveals three types of filaments with a different number of protofilaments with unique morphologies in the VV patient (Fig. 2). The power spectrum of the 2D classes reveals a strong layerline with a central peak on the meridian at 4.8 Å consistent with the presence of a cross-β structure with a 4.8 Å helical rise (Figure S5b, c). The apparent crossover distance of the major class was roughly 80–100 nm with a width of ~ 11 nm, yielding a ~ 0.7–1.0° twist per 4.8 Å rise along the helical axis. We observe three forms of APrP filaments, a doublet, a triplet and quadruplet containing two, three, or four ring-shaped protofilaments, respectively, intertwined into a left-handed helix (Fig. 2b). However, a significantly weaker density is observed in the single bottom (triplet) and two bottom (quadruplet) protofilaments (Fig. 2b), suggesting that these protofilaments are not uniformly assembled. We obtained high-resolution reconstructions of the doublet, triplet and quadruplet filaments at 3.29, 5.50, and 3.13 Å, respectively (Figure S5d). The refined twist value for the doublet was − 0.92°, and for the triplet was − 0.89°, and quadruplet was − 0.94°. The doublet contains two parallel protofilaments sharing a C2 symmetry axis coincident with the helical axis, hereby referred to as Type I (Fig. 2). In the high-resolution reconstruction of the triplet and quadruplet classes, hereby referred to as Type IIa/b, respectively, we observe two identical antiparallel protofilaments offset from the center that appear to be mirrored across the *Y*-axis without true symmetry (Fig. 2). In Type IIa/b, the signals of the weaker protofilaments previously seen in the low-resolution 3D classification (Fig. 2b) are further reduced to a background level (Fig. 2c). In the MV patient, we observe only a doublet form of the APrP filament reconstructed to 3.82 Å resolution with an identical fold to the Type I of the VV patient (Fig. 2) (Figure S5d). The final helical parameters used for each of the reconstructions are shown together with data collection and processing statistics (Table S1).

### APrP fibril structures have a unique spiral protein fold

**Fig. 3:**
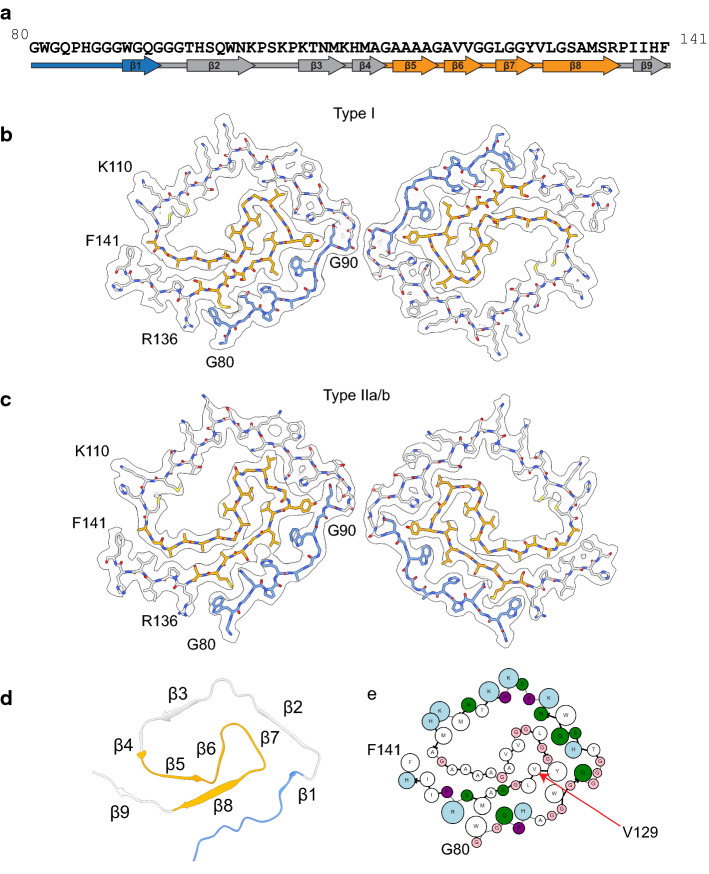
Atomic models of APrP amyloid core derived from cryo-EM reconstructions. APrP protofilament core showing the location of the nine β-strand regions in the protein sequence (**a**). Residues belonging to the octapeptide repeat region (G80-Q91) are indicated in blue. Residues belonging to the hydrophobic domain (A113-S135) are indicated in orange. Cryo-EM map in transparent gray, with atomic model of a single molecule of the APrP filament for Type I (**b**) and Type II (**c**). Ribbon diagram of a single protofilament unit of APrP depicting the β-strand regions (**d**). Cartoon representation of amino acid residues. Hydrophobic (white), positively charged (teal), polar (green), proline (purple), and glycine (pink) residues are highlighted. Valine 129 is indicated by an arrow (**e**)

The protein identity and the sequence register were unambiguously assigned using phenix.sequence_from_map and further validated by findMySequence (Fig. S6a). The high-resolution helical reconstructions of Type I and Type II filaments allowed ab initio atomic modeling of the core of the protofilaments (Fig. S6b, c). Despite the differences in helical organization, Type I and Type II protofilaments are ultrastructural polymorphs, with an identical spiral fold. The spiral protein fold contains 62 residues from G80 to F141 (Fig. 3, Fig. S2a). The sequence contains part of octapeptide repeat 4 (W80-Q83) and all of repeat 5 (P84-Q91), the hydrophobic region (A113-S135), and two interconnecting segments (Q91-M112 and R136-F141) (Fig. 3a). The model has nine β-strands comprising residues 89-WGQ-91 (β1), 95-THSQWN-100 (β2), 106-KTNM-109 (β3), 111-HMA-113 (β4), 115-AAAA-118 (β5), 120-AVV-122 (β6), 125-LGG-127 (β7), 129-VLGSAMS-135 (β8), 138-IIH-140 (β9), with β1, β2, β3, β4, and β9 forming the outer layer and β5, β6, β7, and β8 in the inner layer of the spiral fold. The two layers containing the β1 and the β8 strands engage in a steric zipper between the stretch of residues 81-WGQPHAGGW-89 and 128-YVLGSAMSR-136. Between β4 and β9, there is a group of hydrophobic residues (112–141) (Greek key motif) [46], that differs from the topology previously reported in other PrP structural studies [16, 22, 25, 45, 55]. The C-terminal part of the Greek key arch (residues 128–135) provides the base of the steric zipper between β1 and β8 (Fig. 3d).

The N- and C-termini of the resolved regions of the protomer are exposed on the same side, distal from the protofilament interfaces. Bulky side-chain amino acids are predominantly located on the exterior of the structure, while smaller amino acids in the inner layer form a hydrophobic core (Fig. 3e) explaining the apparent thin densities in the inner region and thick densities in the outer layer in the electron densities (Fig. 2c). In addition, there is a cluster of exposed positively charged residues from K101 to H111 and a large hydrophobic cavity formed by the amino acid sequence between residues P102 and G124 including the hydrophobic region 113-AGAAAAGA-120 (Fig. 3e, Figure S7a).

In Type I and Type II filaments, the interactions at the interfaces of the protofilaments are mediated by the GGG motif in residues G92-G94, although the exact interacting residues vary slightly (Fig. 3b, c). In Type I filaments, the GGG motifs from the two parallel protofilaments related by two-fold symmetry are nearly perfectly aligned in opposite directions to allow direct interaction between G92/G94, G93/G93, and G94/G92 pairs, respectively. In Type II filaments, the GGG motifs from the two antiparallel protofilaments are slightly offset by one amino acid, and pack between G92 of one protofilament and G92/G93 of the other protofilament.

Several types of interactions contribute to the stability of the fibril assembly. The chief interactions are the backbone hydrogen bonds of the cross-β sheets and the parallel in-register stacking of the amino acid residues along the fibril axis. Furthermore, each protomer layer is not planar, but spans three layers with a *Z* height variation of ~ 7–8 Å in both Type I and Type II fibrils. This allows each layer i to interact with its immediate layers i ± 1 as well as layers i ± 2 that are beyond its immediate vicinity (Figure S7b, c). The non-planarity, due to preferred residue pair interactions during fibril formation, results in a small tilt of the layer with respect to the fibril axis. The non-planar nature of each layer contributes to the rigidity of the fibrils while introducing a subtle polarity to the fibrils because of structural differences between the two fibril ends. Moreover, these protofilaments form dimers, trimers, and tetramers thereby enhancing the stability of the fibrils.

### Additional densities associated with the filaments

Multiple extra densities at identical positions in Type I and II filaments were observed, with large solvent-exposed densities near residue K104 and near K110/H111 (Fig. S7d). Mass spectrometry analysis of the samples did not reveal modifications at K104; however, peptides containing acetylated K110 and acetylated and formylated H111 were found (Fig. S3). Two small solvent-exposed densities in between the two large densities near residues K106 and N108 and four small fragments of electron density in the hydrophobic cavity near residues T107, M109, M112, and V121 were also observed. Mass spectrometry analysis shows acetylation of T107, and oxidation and sulfonation of M112; however, considering the discrete positions with clear gaps from the protein densities, these four small extra densities are likely hydrophobic host factors.

## Discussion

Previous studies have shown that amyloid deposits in the brain of patients affected by GSS (F198S) are made of low molecular weight PrP fragments which are derived from the central region of human PrP and have ragged N- and C-termini. In addition, fragments corresponding to the amino and carboxyl domains of full-length PrP have been detected by mass spectrometric analysis and by immunohistochemistry using antibodies to the N- and C-terminal domains [4, 15, 28, 40, 41].

The central goal of this study was to determine the atomic structure of the filaments of human APrP isolated from the brains of two GSS (F198S) patients. The immunohistochemical analysis carried out in the GSS (F198S) patients shows that the core of the amyloid plaques is immunoreactive with antibodies against epitopes located in the central region of PrP, while the periphery of the amyloid plaque cores is strongly immunoreactive with antibodies to N- and C-terminal domains. The water extracted PrP from cerebellum, analyzed by western blot, shows an identical pattern of migration in the GSS (F198S) patients with the 129 VV and 129 MV haplotypes. The results of the mass spectrometric analysis provide insights on the complexity of the APrP composition, with a majority of peptides having a ragged N-terminus (around amino acid 80) and a ragged C-terminus (between amino acids 149–170).

**Fig. 4:**
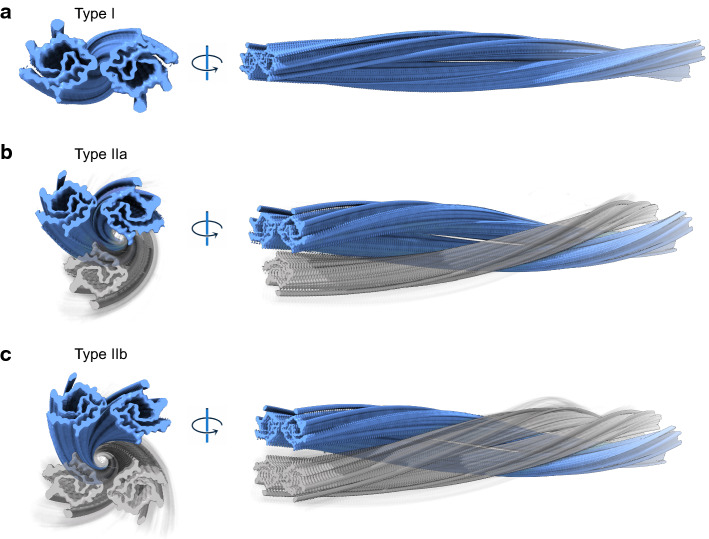
Hypothetical models of Type I, Type IIa, and Type IIb. High-resolution reconstructions are depicted in blue. Low occupancy/unresolved density filaments for Type IIa and Type IIb are colored in transparent gray

Cryo-EM of water extracted PrP shows amyloid filaments that are polymorphic, consisting of two, three or four intertwined protofilaments in patient 1, homozygous at codon 129 (VV) (Fig. 4). The antiparallel organization of the protofilaments in the Type II doublets seems to be uncommon among amyloid structures (Figure S7c) [9, 16, 18, 35]. Each protofilament exhibits a common double spiral protein fold containing sixty-two amino acids, encompassing residues G80 to F141 of PrP. The core begins in the fourth octapeptide repeat and contains the fifth octapeptide repeat, the hydrophobic region and the small β-strand (amino acids 128–131) of PrP^c^ [53]. The theoretical molecular weight (average) of a peptide spanning residues G80 to F141 of PrP is ~ 6.210 kDa, suggesting that additional N- and C-terminal residues may be involved forming a “fuzzy coat”, accounting for the experimental higher molecular weight bands (~ 8 kDa and higher) observed on western blots. The data currently presented are in complete agreement with previous reports indicating that the core of APrP associated with GSS (F198S) encompasses residues 70 to 150 [4, 40]. Additional densities were identified, specifically at the solvent-exposed sidechains of K104 and near K110/H111. In addition, four small electron densities were observed in the hydrophobic cavity near residues T107, M109, M112, and V121. Mass spectrometric analysis shows the presence of PTMs at amino acids K110 (acetylation) and H111 (acetylation and formylation) suggesting that the PTMs may be responsible for some of the densities observed by cryo-EM, while anionic host factors could account for the remaining densities [24]. A large number of proteins co-purified with APrP, the APrP “interactome”, composed of a variety of proteins involved in the cytoskeleton and cytosol, focal adhesion membranes, extracellular exosomes, DNA replication, telomere organization, cytoplasmic translation, nucleosome assembly and RNA binding. The amyloid-associated proteins apolipoprotein E (apoE), apolipoprotein J (apoJ or Clusterin) and serum amyloid P component (SAP or Pentraxin 2), which are co-deposited with amyloid proteins, form part of the APrP “interactome” [30]. In addition, proteins that have been previously reported in association with PrP deposits were also present, like Vimentin, complement C1q subunits, apolipoprotein D (apoD), glial fibrillary acidic protein (GFAP), cofilin-1 and destrin [27].

The methionine/valine polymorphism at codon 129 in human *PRNP* may be a modifier of the clinical and pathologic presentation of prion diseases [5, 6, 10, 14]. In view of the fact that the F198S *PRNP* mutant allele is in cis with valine 129, either valine homozygosity or methionine/valine heterozygosity at position 129 of PrP can be found in the GSS (F198S) mutation carriers. On average, in GSS (F198S) patients homozygous for valine at codon 129, the onset of clinical signs occurs approximately 10 years earlier than in the heterozygous methionine/valine patients [6]. The limited availability of retrospective anamnestic data for the two patients did not allow to determine their respective age at the onset of clinical symptoms.

**Fig. 5:**
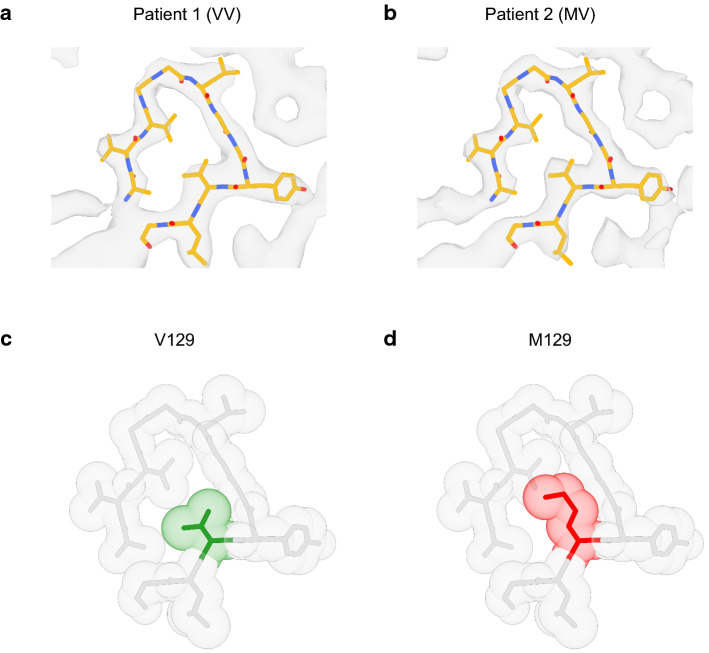
Structural analysis of the M/V 129 residue region of APrP. Type I atomic model superimposed with the map of APrP Type I from patient 1 (129 VV) (**a**) and patient 2 (129 MV) (**b**). Zoomed-in view of the structure around residue 129. The atoms are shown as transparent spheres with their radii equal to the Van der Waals radii. The 129 V residue (green) is tightly packed (**c**) with little space to accommodate a bulkier 129 M residue (red) (**d**)

Valine 129 is buried in the amyloid core, forming the β8 strand (129-VLGSAMS-135) in the hydrophobic Greek key motif (Fig. 3e), where it is strongly protected from solvent exchange [36, 55]. In the GSS (F198S) APrP protofilaments, the side-chain density of valine is tightly packed leaving little space for a bulky methionine side-chain (Fig. 5), suggesting that peptides containing methionine at 129 may affect fibril formation. In vitro studies have shown that substitution of methionine 129 by asparagine 129 completely inhibited both spontaneous and seeded aggregation, while the presence of valine 129 resulted in a strong enhancement of aggregation [36, 55]. Thus, substitution of valine 129 by methionine 129 in APrP fibrils in patients heterozygous at codon 129 could affect protein aggregation in vivo and result in a delay of age at onset as seen in heterozygous (methionine/valine) GSS (F198S) patients [6]. The cryo-EM structures of APrP obtained from patient 1, a symptomatic individual homozygous for valine at codon 129, showed amyloid filaments that are polymorphic, consisting of two, three or four intertwined protofilaments. In patient 2, an individual heterozygous MV at codon 129, only a doublet protofilament species is observed with an identical double spiral fold to the Type I filament from patient 1. In addition, this fold contains the same extra densities as the folds from patient 1. The absence of the triplet and quadruplet forms of the APrP filaments in patient 2 may be due to variations in the extraction process or the dispersion procedure used for cryo-EM imaging. It is also possible that other intrinsic biochemical conditions in the brains of the patients could play a role in the formation of the APrP triplet and quadruplet aggregates seen in patient 1. In the Type I structure of patient 2, the 129 residue position’s map density is more consistent with the presence of valine than a methionine residue (Fig. 5a, b). This may suggest that a PrP peptide with methionine at 129 is less likely to be incorporated into the APrP filaments likely due to steric hindrance (Fig. 5c, d). Peptides with methionine at 129 may interfere with fibril growth and potentially be responsible for the delay of age at onset in heterozygous (valine/methionine) GSS (F198S) patients.

This study has identified for the first time the structure of APrP filaments from human brain. Together with the recently reported structures of Aβ filaments in AD [52], the APrP filaments are the sole structures described in human diseases characterized by a brain amyloidosis which coexists with Tau aggregates and NFTs. Additional research is needed to determine whether a common mechanism may trigger aggregation of Tau, resulting in Tau filaments with identical structure at their core [18, 35]. Recently, Tau PET scanning has enabled correlation of Tau localization with magnetic resonance imaging in GSS patients in vivo [31]; however, due to a lack of APrP-specific ligands, prion imaging has not yet been possible. The structural data of APrP in this study open new avenues for generating novel APrP ligands for both diagnosis and therapeutic targets.

## Supplementary Information

Below is the link to the electronic supplementary material.

Supplementary file1 (PDF 57244 KB)
