# Supplementary material for: Cryo-EM structures of prion protein filaments from Gerstmann–Sträussler–Scheinker disease
Source: Acta Neuropathol. 2022 Jul 12;144(3):509–20. doi: 10.1007/s00401-022-02461-0 (PMC9381446; doi:10.1007/s00401-022-02461-0)
Supplement: Supplementary file 1 — Supplementary file1 (PDF 57244 KB) [file 401_2022_2461_MOESM1_ESM.pdf]

Cryo-EM structures of Prion protein filaments from Gerstmann-Sträussler-Scheinker disease

Grace I. Hallinan[1,*], Kadir A. Ozcan[2,*], Md Rejaul Hoq[2], Laura Cracco[1], Frank S. Vago[2], Sakshibeedu R. Bharath[2], Daoyi Li[2], Max Jacobsen[1], Emma H. Doud[3], Amber L. Mosley[3,4], Anllely Fernandez[1], Holly J. Garringer[1], Wen Jiang[2,#,+], Bernardino Ghetti[1,#,+], Ruben Vidal[1,5,#,+]

[1]Department of Pathology and Laboratory Medicine, Indiana University School of Medicine, Indianapolis, IN 46202, USA
[2]Department of Biological Sciences, Markey Center for Structural Biology, Purdue University, West Lafayette, IN 47906, USA
[3]Center for Proteome Analysis and Center for Computational Biology and Bioinformatics, Indiana University School of Medicine, Indianapolis, IN 46202, USA
[4]Biochemistry and Molecular Biology, Indiana University School of Medicine, Indianapolis, IN 46202, USA
[5]Stark Neurosciences Research Institute, Indiana University School of Medicine, Indianapolis, IN 46202, USA

Supplementary Materials and Methods

Human Tissues. Human brain samples and clinical records were collected before the research was proposed to the institutional review board to determine whether the research is exempt (Category 4) under an IRB-approved autopsy protocol. Subjects cannot be identified, directly or through identifiers linked to the subjects. All work using human materials or samples derived from humans was handled at BSL2. Both sites, IUSM and Purdue University are approved to work at BSL2.

Experimental model and subject details.

Clinical history. Two Patients, carriers of the *PRNP* F198S mutation, were members of a large kindred, whose pedigree has been traced back to the year 1750.

GSS (F198S) case 1. The patient had not completed a high school education. According to the patient's next-of-kin, the disease manifested itself with neuropsychiatric symptoms that could be detected by a physician when the patient was 49 or 50 years old. Anxiety, confusion, paranoid ideation with hallucinations were reported along with "shakiness of hands and legs". A few months later, at the first neurological visit it was recorded that the patient was right-handed, had been aware of the hereditary disease in the family and had requested genetic counseling. The informant reported a one-year history of gradually progressive memory and language problems and a six-month history of difficulties with judgment and reasoning, changes in personality, irritability, withdrawal, sadness, socially inappropriate behavior, and agitation. On neurological and neuropsychological examinations, bradykinesia, an abnormal gait, and a slight tremor were recorded, while the global Clinical Dementia Rating (CDR) was 0.5, indicating mild

global impairment, with mild impairment (a 0.5 rating) in the memory, orientation, judgement and problem-solving, community affairs, and home and hobbies domains (CDR Sum of Boxes = 2.5), and moderate impairment (a 1.0 rating) in the behavior, comportment, and personality domains of the supplemental CDR. The neuropsychiatric inventory (NPI) indicated the presence of agitation, depression, anxiety, disinhibition, irritability, nighttime behaviors, and problems with appetite with severity scores predominantly in the mild range. The functional assessment scale (FAS) indicated mild impairment in daily functioning (total score = 6). The neuropsychological battery revealed mild impairments in efficiency of new learning (Selective Reminding Test sum recall = 88/144, delayed recall = 5/12), verbal fluency (Controlled Oral Word Association Test (COWA) = 23), response inhibition (Stroop Color-Word raw score = 21), complex sequential tracking (Trail Making Part B = 144 seconds, Wechsler Adult Intelligent Scale (WAIS) Digit Symbol raw score = 24), and manual motor speed (finger taps in 10 seconds right hand = 38, left hand = 36). The Mini-Mental State Examination score was 26/30. The Gordon Diagnostic System (GDS) was within normal limits. The consensus diagnosis was mild dementia due to GSS. One year later, at age 52, during the follow-up assessment, the following was reported: presence of delusions and advanced deterioration of memory, language, judgment, reasoning, and personality. On neurological examination, there was evidence of Parkinsonism, frequent falls, and gait disturbances. The global CDR had progressed to 1.0, indicating moderate impairment, with mild impairment (a 0.5 rating) in memory and orientation domains, mild to moderate impairment (a 1.0 rating) in judgement and problem-solving, community affairs, and personal care domains, and severe impairment (a 3.0 rating) in the home and hobbies

domain (CDR Sum of Boxes = 7.0). The NPI included delusions in addition to the problems noted on the previous visit, with most symptoms rated as moderate in severity. The FAS total score had progressed to 13, with need for assistance in shopping, simple meal preparation and cooking, and traveling. The second neuropsychological examination revealed mild to moderate declines in new learning, memory, executive cognitive function, psychomotor speed, and manual motor skills (Rey Auditory Verbal Learning Test sum recall = 22/75, delayed recall = 3/15; Craft Story delayed recall 4/25, Benson Figure delayed recall raw score 8/17, Letter F and L fluency = 11, Trail Making Part B = 357 seconds, WAIS Digit Symbol raw score = 20, finger taps in 10 seconds right hand = 24, left hand = 25). The Montreal Cognitive Assessment (MoCA) score was 18/30 and GDS was within normal limits. The consensus diagnosis was dementia due to GSS. The [^{18}F] flortaucipir PET and the structural magnetic resonance imaging (MRI) scans were completed at this visit [29]. Patient died while at hospice care at age 53.

GSS (F198S) case 2. Information on the patient is limited. The deceased father was affected by GSS. Patient was examined neuropsychologically at age 31 during an eye movement study. The test results indicated mild cognitive and motor dysfunction and no eye movement abnormalities. During the following years, the patient lived in Texas and was not regularly seen by neurologists at Indiana University. At age 35, the patient suffered low back and neck pain. A neurological examination revealed diffuse hyperreflexia and an abnormal gait related to spasticity of the lower extremities. A computerized tomography (CT) scan of the lumbar spine showed a disc herniation at L5-S1. At age 48, during a neurological examination, it was reported that during the

previous two years, the patient’s speech had become somewhat slurred and the gait less stable. Some difficulty with word finding was also evident. In view of the long history of spasticity, it was thought that signs and symptoms of GSS might have been potentially masked. Therefore, a genetic test was carried out, revealing the *PRNP* F198S mutation. At age 53, the patient’s GSS symptoms had progressed and admission to a Care Center was necessary. Patient had multiple medical conditions, including chronic obstructive pulmonary disease and hypertension. Bimalleolar fractures were also reported. The patient could not follow commands, had severe sleepiness, and visual hallucinations. The patient deteriorated and died at age 54.

Mass spectrometry sample preparation. Samples were diluted in 8 M urea, 50 mM Tris-HCl pH 8.5 (100 µl), reduced with 5 mM Tris (2-carboxyethyl) phosphine hydrochloride (TCEP-HCl) for 30 min at 37°C and alkylated with 10 mM chloroacetamide at RT in the dark, for 30 min. Samples were digested in two steps with LysC/trypsin (Promega). After overnight trypsin digestion in 2 M Urea, the samples were applied to Pierce detergent removal spin columns (Thermo Scientific) and then desalted on SepPak 18 cartridge (Waters Corporation) washed with 1 mL of 0.1% trifluoroacetic acid (TFA), eluted in 600 µL of 70% Acetonitrile (ACN)/0.1 % formic acid (FA)), and dried by speed vac.

Liquid chromatography (LC) with tandem mass spectrometry (MS). Samples were reconstituted in 50 µL of 0.1 % FA and 7 µL were injected on an Easynano LC1200 coupled with Aurora 25cm column (IonOptiks) in sonation column oven (40 °C) on an Eclipse Orbitrap mass spectrometer (Thermo Fisher Scientific). Peptides were eluted on a 115 minute gradient from 5-35 % B, increasing to 95% B over 10 minutes, and decreasing to 5% B for 5 minutes (Solvent A: 0.1% FA; Solvent B: 80% ACN, 0.1% FA). The instrument was operated with FAIMS pro 4 CVs (-30, -45, -55, -65 V), positive mode, 0.6 second cycle time per CV with APD

and Easy-IC on. Full scan 400-1500 m/z with 60000 resolution, standard AGC and auto max IT, 40% RF lens, 5e4 intensity threshold, charge states 2-8, 30 sec dynamic exclusion with common settings. MS2 parameters of 1.6 m/z quadrupole isolation, 30% fixed HCD CE, 15000 orbitrap resolution, standard AGC and dynamic IT.

MS data analysis. Raw files were loaded into PEAKS X Pro Studio 10.6 Build 20201221 (Bioinformatics Solutions) precursor ion tolerance was 10 ppm 0.02 Da, specific trypsin digestion, database searches of the reviewed Uniprot_Swissprot Homo sapiens database and common contaminants (20437 entries) with variable post-translational modifications (PTMs). PEAKS PTM and SPIDER searches were enabled to search all de novo peptides above 15% score for over 300 potential PTMs and mutations. A 0.1 % peptide FDR cutoff (-10lgP≥21.8), PTM Ascore > 10, mutation ion intensity >1 and de novo only score > 80% were applied to the data followed by PEAKS LFQ analysis. Raw and searched data are available at ProteomeXchange [47]. The bio-informatic analysis Gene Ontology of identified proteins was done by DAVID Bioinformatics Resources 6.8 (https://david.ncifcrf.gov). pValue was represented as -Log10. The Venn diagram was generated using BioVenn (http://www.biovenn.nl/).

Supplementary Figure Legends

Figure S1. Members of the GSS F198S family pedigree. Affected members (filled symbols) and obligate escapees (open symbol) are shown. Roman numbers indicate different generations. Individual VI(7) (red square, patient 1) is a descendent of affected case V(10), and individual VII(11) (green square, patient 2) is a descendent of affected case VI(4).

Figure S2. Representative diagram of Prion protein (253 aa). Epitopes of specific antibodies (abs) used in the study are indicated (23-40, N-terminus; 95-108 and 106-110 (3F4), middle region; 220-231, C-terminus). The spiral protein fold in GSS (F198S) contains 62 residues from G80 to F141 (a). Negative stain EM images of isolated APrP filaments from the cerebellum of patient 1 (b). Western blots of APrP isolated from cerebellum of GSS (F198S) patient 1 (129VV) and patient 2 (129MV) using antibody 3F4 (c). Homozygous valine 129 (129VV) and heterozygous methionine/valine129 (129MV). Molecular masses are in kDa.

Figure S3. MS of isolated APrP from the cerebellum of patient 1 (a) and patient 2 (b). The MS shows ragged N- and C- termini, with the majority of the peptides starting near (or just before) amino acid G80 and ending near amino acids Y150 or V160. Only peptides not generated by trypsin are indicated. Peptide start (<) and peptide end (>). Number of peptides observed beginning/ending at the particular amino acid (black, 1-4; green, 5-9; red, more than 10). PTMs shown are phosphorylation (yellow), deamidation (blue), acetylation (purple) and oxidation (orange).

Figure S4. APrP interactome. Proteins identified by MS that co-purify with amyloid deposits from patient 1 (129VV) and patient 2 (129MV) were analyzed. A total of 882 proteins were present in both samples (a). These proteins were significantly clustered in three different groups; biological processes (b), cellular components (c) and molecular functions (d). The 4 most significant Gene Ontology (GO) terms with -log10 (P-value) are indicated. GO bar plots were made using DAVID Bioinformatics Resources 6.8.

Figure S5. Representative cryo-EM images of aggregated APrP filaments (left) and dispersed filaments (right) from patient 1 (a). Power spectra of Type I and Type II 2D class averages. The 4.8 Å layer line indicates that the APrP filaments have a cross-beta structure (b). Side-view of the cryo-EM 3D reconstructions of Type I and Type II filaments (c). Fourier shell correlation (FSC) curves for two independently-refined cryo-EM half-maps of Type I and II filaments. The FSC threshold of 0.143 is shown as a red dashed line (d). Scale bar, 100 nm (a), 10 Å (c).

Figure S6. Analyses using phenix.sequence_from_map (left) and findMySequence (right) confirm the identity of PrP in the density maps (a). Representative regions of the APrP atomic model built into the cryo-EM maps (white mesh) (b). FSC curves for the refined atomic model against the cryo-EM density map for Type I (left) and Type II (right). The FSC threshold of 0.5 is shown as a red dashed line.

Figure S7. Surface charge depiction of the APrP atomic model. Positively charged (blue), negatively charged (red) and hydrophobic (white) regions are highlighted (a). Secondary structure elements of the APrP spiral fold depicted as 5 molecules stacked upon one another of the Type I (b) and the Type II (c) atomic models. Z axis plot of a single layer of the APrP atomic models of Type I (b) and Type II (c). The X axes depicts residue number with sequence below. Y axes represents the Z position along the helical axes of the α-carbon. Cryo-EM maps, depicted as central slices. Arrows point to extra

densities not modeled by protein. Putative solvent exposed densities near K110, H111 and K104 (cyan), and hydrophobic core densities (orange).

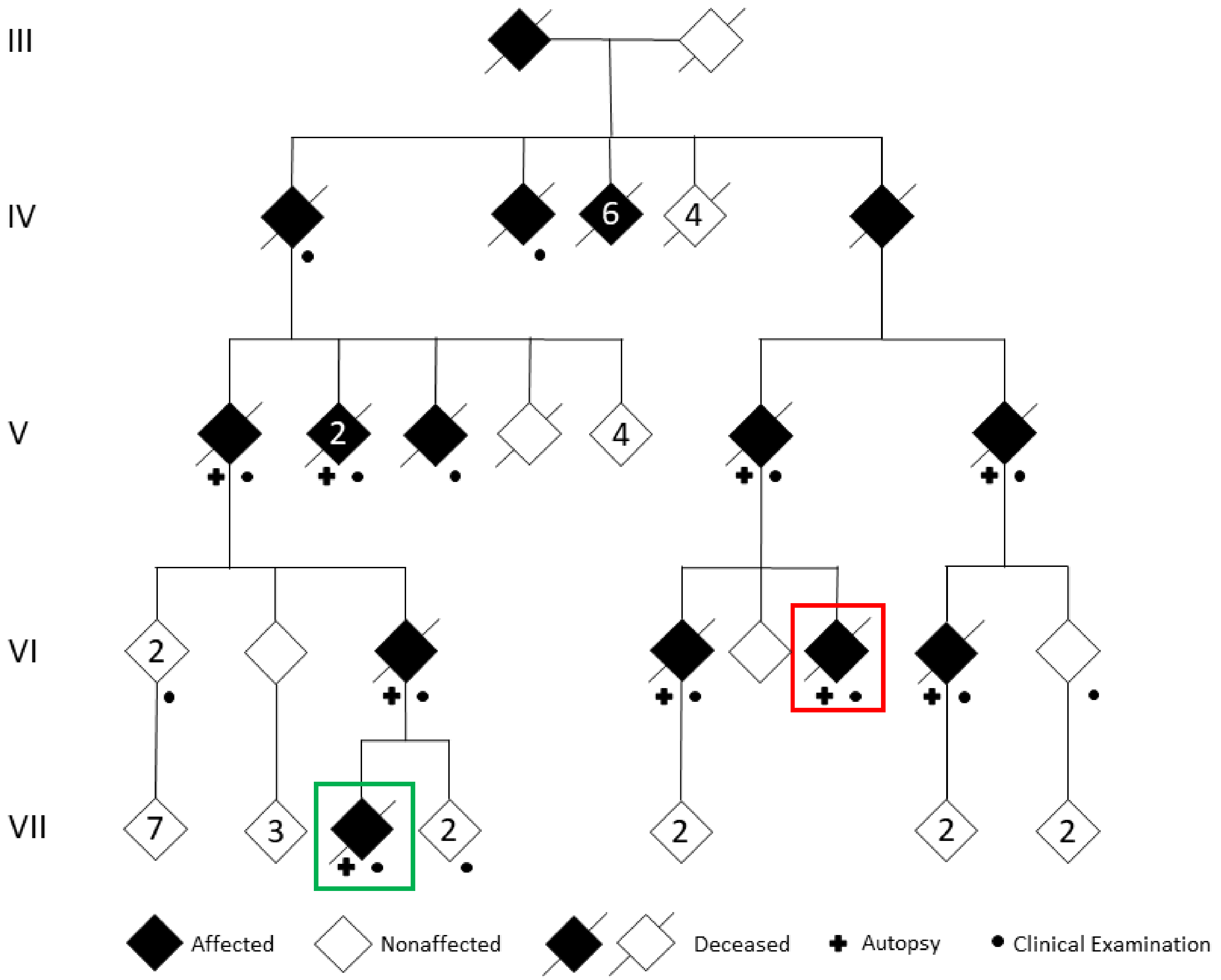
III
IV
V
VI
VII
6
4
2
4
2
7
3
2
2
2
2
Affected
Nonaffected
Deceased
Autopsy
Clinical Examination

SFig 2

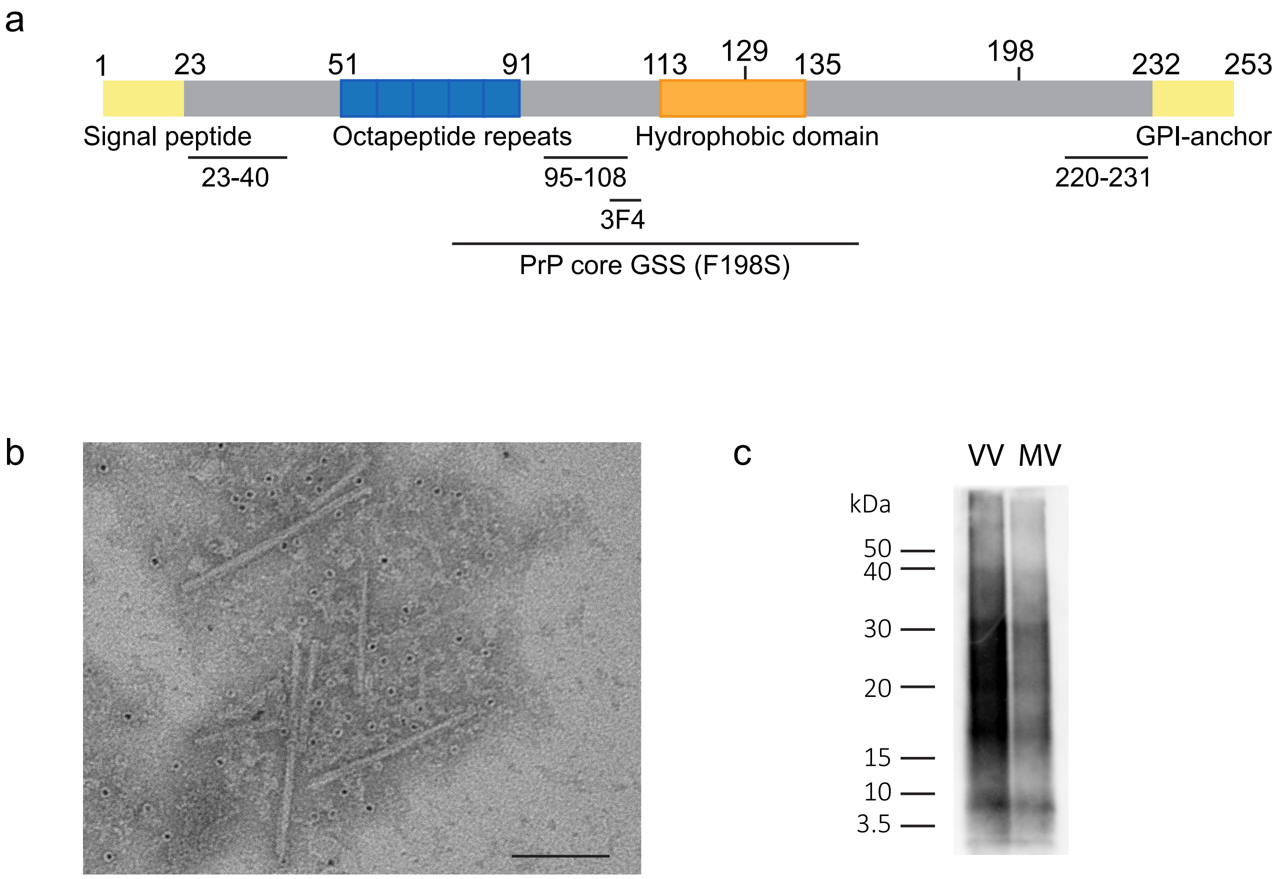
a
1
23
51
91
113
129
135
198
232
253
Signal peptide
Octapeptide repeats
Hydrophobic domain
GPI-anchor
23-40
95-108
220-231
3F4
PrP core GSS (F198S)
b
c
VV MV
kDa
50
40
30
20
15
10
3.5

SFig 3

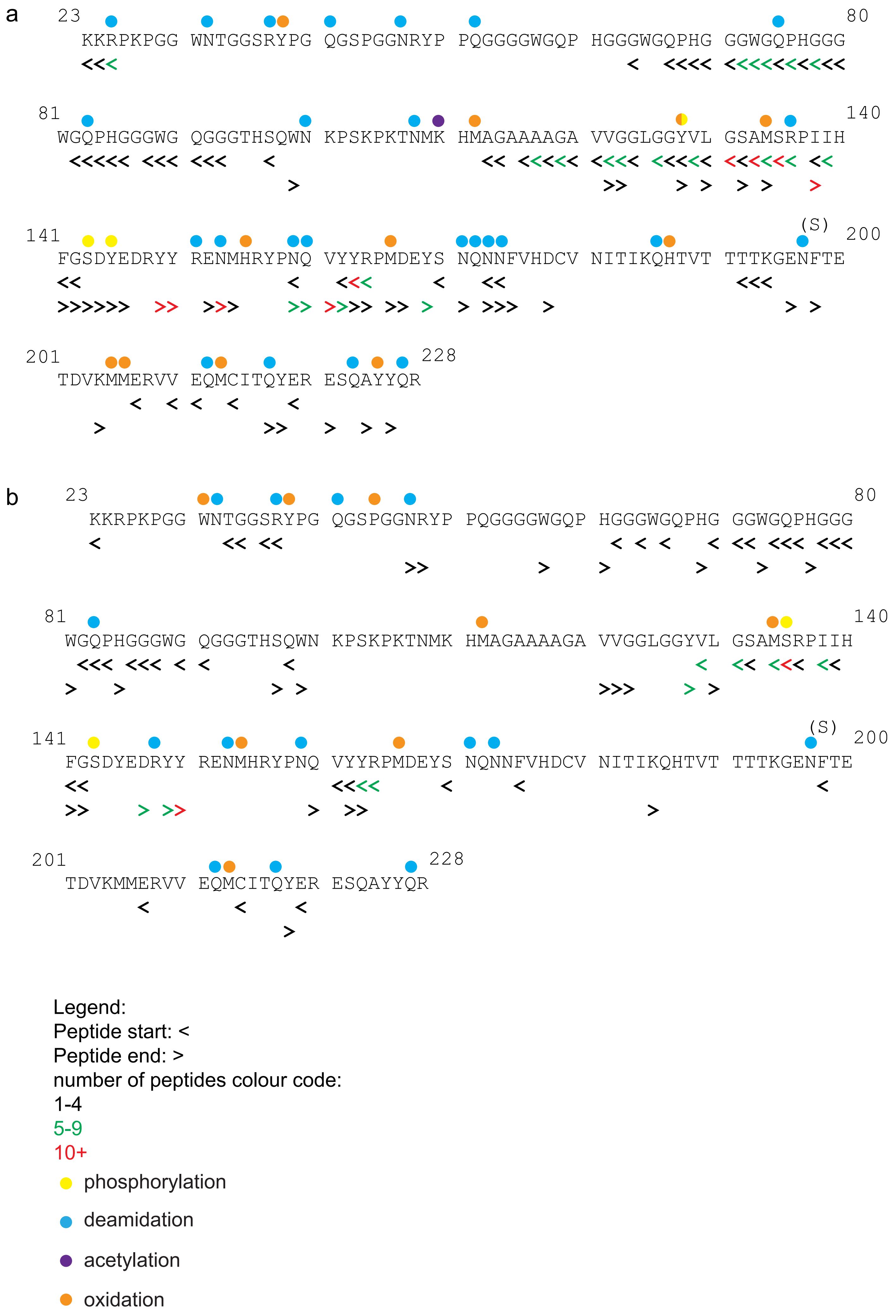
a
23
KKRPKPGG WNTGGSRYPG QGSPGGNRYP PQGGGGWGQP HGGGWGQPHG GGWGQPHGGG
80
81
WGQPHGGGWG QGGGTHSQWN KPSKPKTNMK HMAGAAAAGA VVGGLGGYVL GSAMSRPIIH
140
141
FGSDYEDRYY RENMHRYPNQ VYYRPMDEYS NQNNFVHDCV NITIKQHTVT TTTKGENFTE
(S)
200
201
TDVKMMERVV EQMCITQYER ESQAYYQR
228
b
23
KKRPKPGG WNTGGSRYPG QGSPGGNRYP PQGGGGWGQP HGGGWGQPHG GGWGQPHGGG
80
81
WGQPHGGGWG QGGGTHSQWN KPSKPKTNMK HMAGAAAAGA VVGGLGGYVL GSAMSRPIIH
140
141
FGSDYEDRYY RENMHRYPNQ VYYRPMDEYS NQNNFVHDCV NITIKQHTVT TTTKGENFTE
(S)
200
201
TDVKMMERVV EQMCITQYER ESQAYYQR
228
Legend:
Peptide start: <
Peptide end: >
number of peptides colour code:
1-4
5-9
10+
phosphorylation
deamidation
acetylation
oxidation

SFig 4

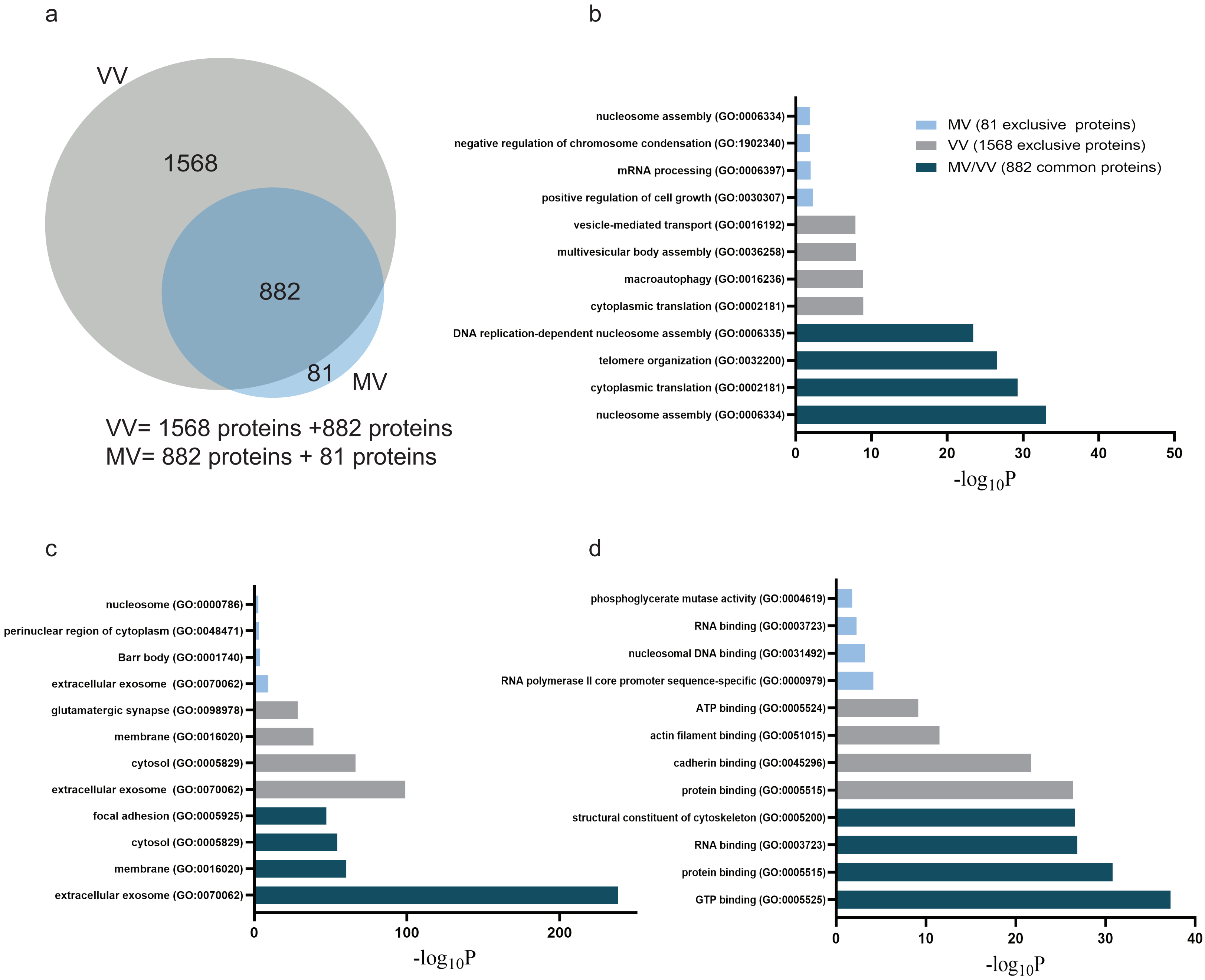

a
VV
1568
882
81
MV
VV= 1568 proteins +882 proteins
MV= 882 proteins + 81 proteins
b
MV (81 exclusive proteins)
VV (1568 exclusive proteins)
MV/VV (882 common proteins)
nucleosome assembly (GO:0006334)
negative regulation of chromosome condensation (GO:1902340)
mRNA processing (GO:0006397)
positive regulation of cell growth (GO:0030307)
vesicle-mediated transport (GO:0016192)
multivesicular body assembly (GO:0036258)
macroautophagy (GO:0016236)
cytoplasmic translation (GO:0002181)
DNA replication-dependent nucleosome assembly (GO:0006335)
telomere organization (GO:0032200)
cytoplasmic translation (GO:0002181)
nucleosome assembly (GO:0006334)
0
10
20
30
40
50
-log10P
c
nucleosome (GO:0000786)
perinuclear region of cytoplasm (GO:0048471)
Barr body (GO:0001740)
extracellular exosome (GO:0070062)
glutamatergic synapse (GO:0098978)
membrane (GO:0016020)
cytosol (GO:0005829)
extracellular exosome (GO:0070062)
focal adhesion (GO:0005925)
cytosol (GO:0005829)
membrane (GO:0016020)
extracellular exosome (GO:0070062)
0
100
200
-log10P
d
phosphoglycerate mutase activity (GO:0004619)
RNA binding (GO:0003723)
nucleosomal DNA binding (GO:0031492)
RNA polymerase II core promoter sequence-specific (GO:0000979)
ATP binding (GO:0005524)
actin filament binding (GO:0051015)
cadherin binding (GO:0045296)
protein binding (GO:0005515)
structural constituent of cytoskeleton (GO:0005200)
RNA binding (GO:0003723)
protein binding (GO:0005515)
GTP binding (GO:0005525)
0
10
20
30
40
-log10P

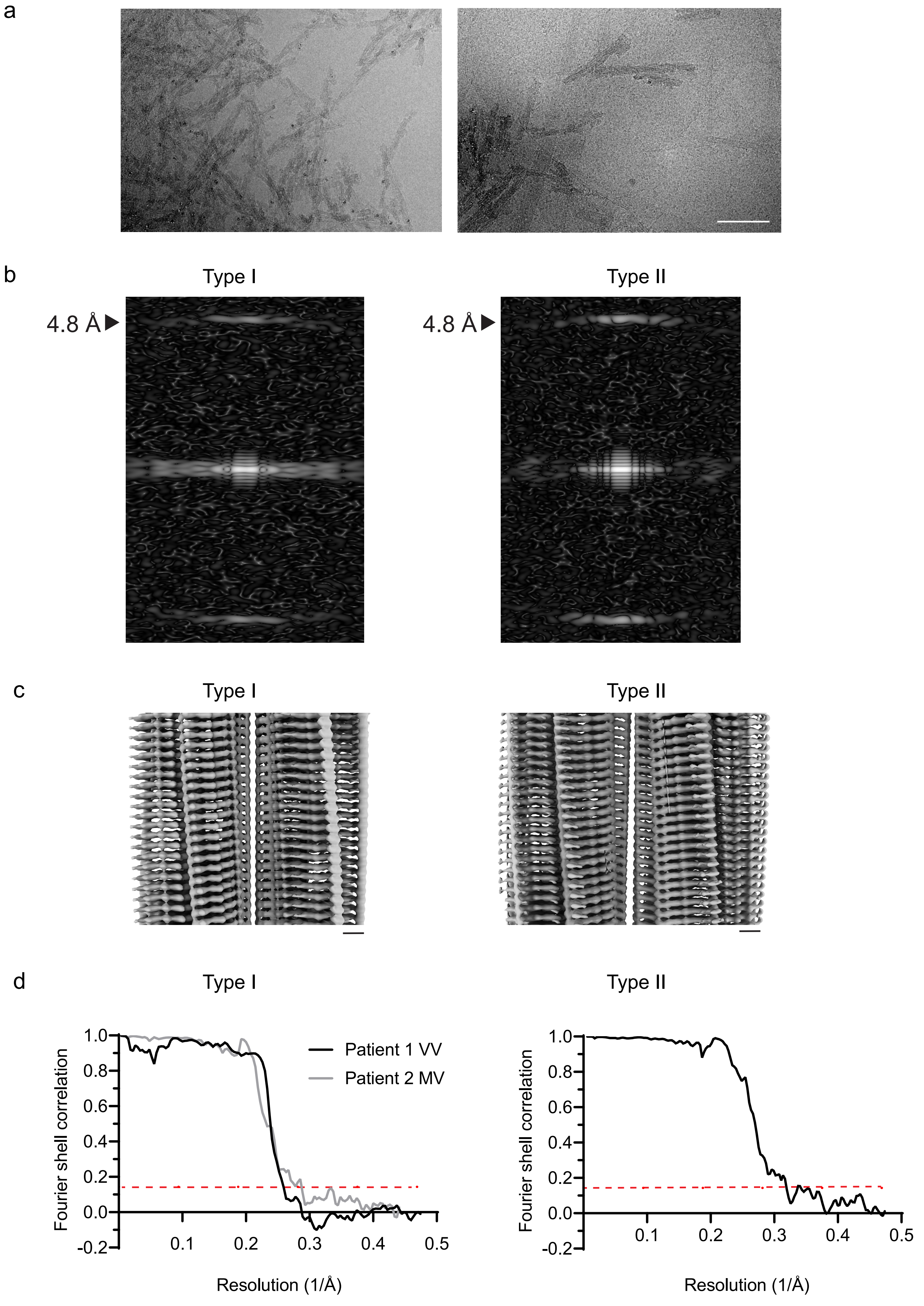
a
b
Type I
Type II
4.8 Å
4.8 Å
c
Type I
Type II
d
Type I
Type II
Patient 1 VV
Patient 2 MV
Fourier shell correlation
Resolution (1/Å)
1.0
0.8
0.6
0.4
0.2
0.0
-0.2
0.1
0.2
0.3
0.4
0.5
Fourier shell correlation
Resolution (1/Å)

SFig 6

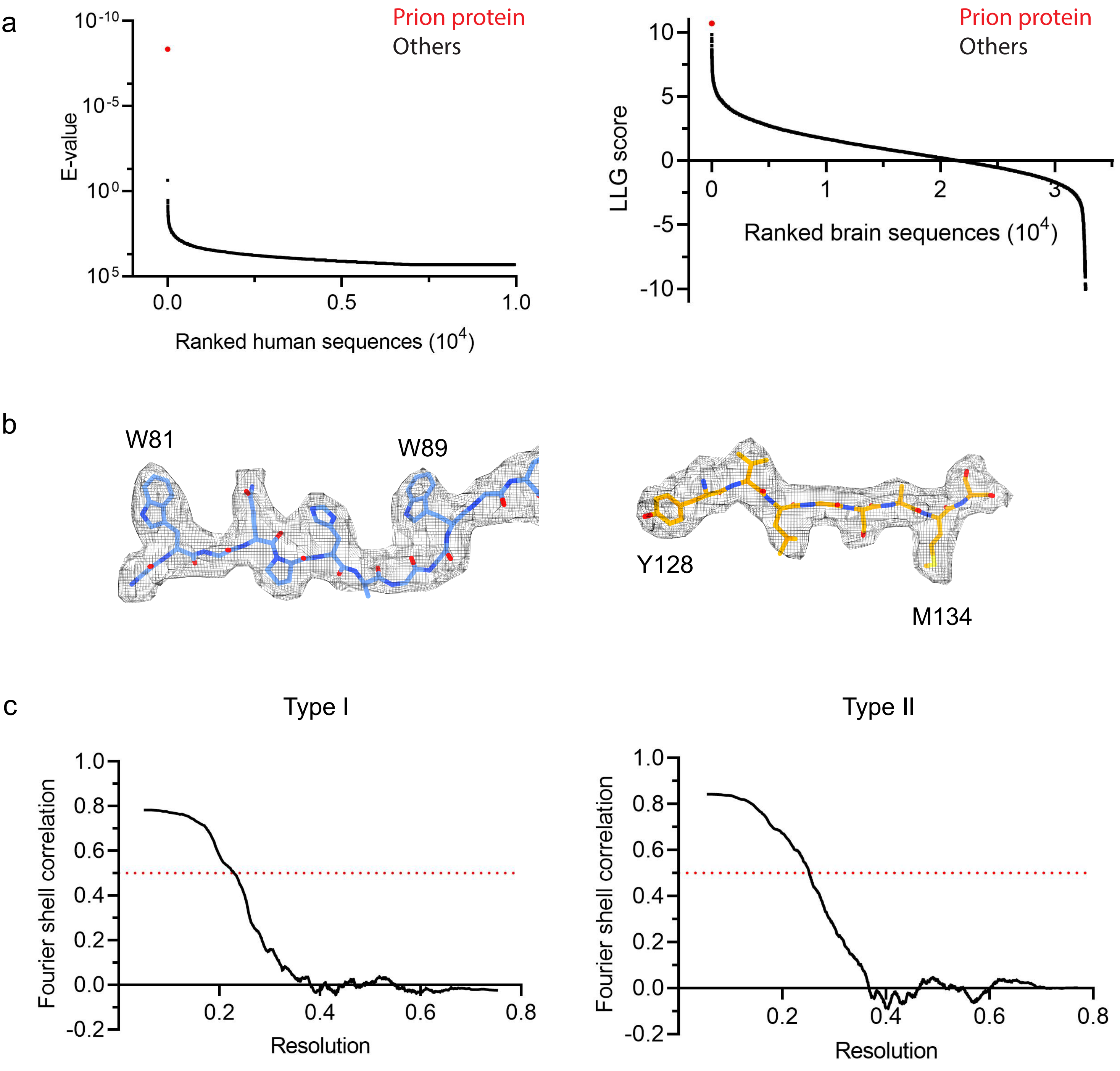
a
Prion protein
Others
E-value
10-10
10-5
10⁰
10⁵
0.0
0.5
1.0
Ranked human sequences (10⁴)
Prion protein
Others
LLG score
10
5
0
-5
-10
0
1
2
3
Ranked brain sequences (10⁴)
b
W81
W89
Y128
M134
c
Type I
Fourier shell correlation
Resolution
1.0
0.8
0.6
0.4
0.2
0.0
-0.2
0.2
0.4
0.6
0.8
Type II
Fourier shell correlation
Resolution

SFig 7

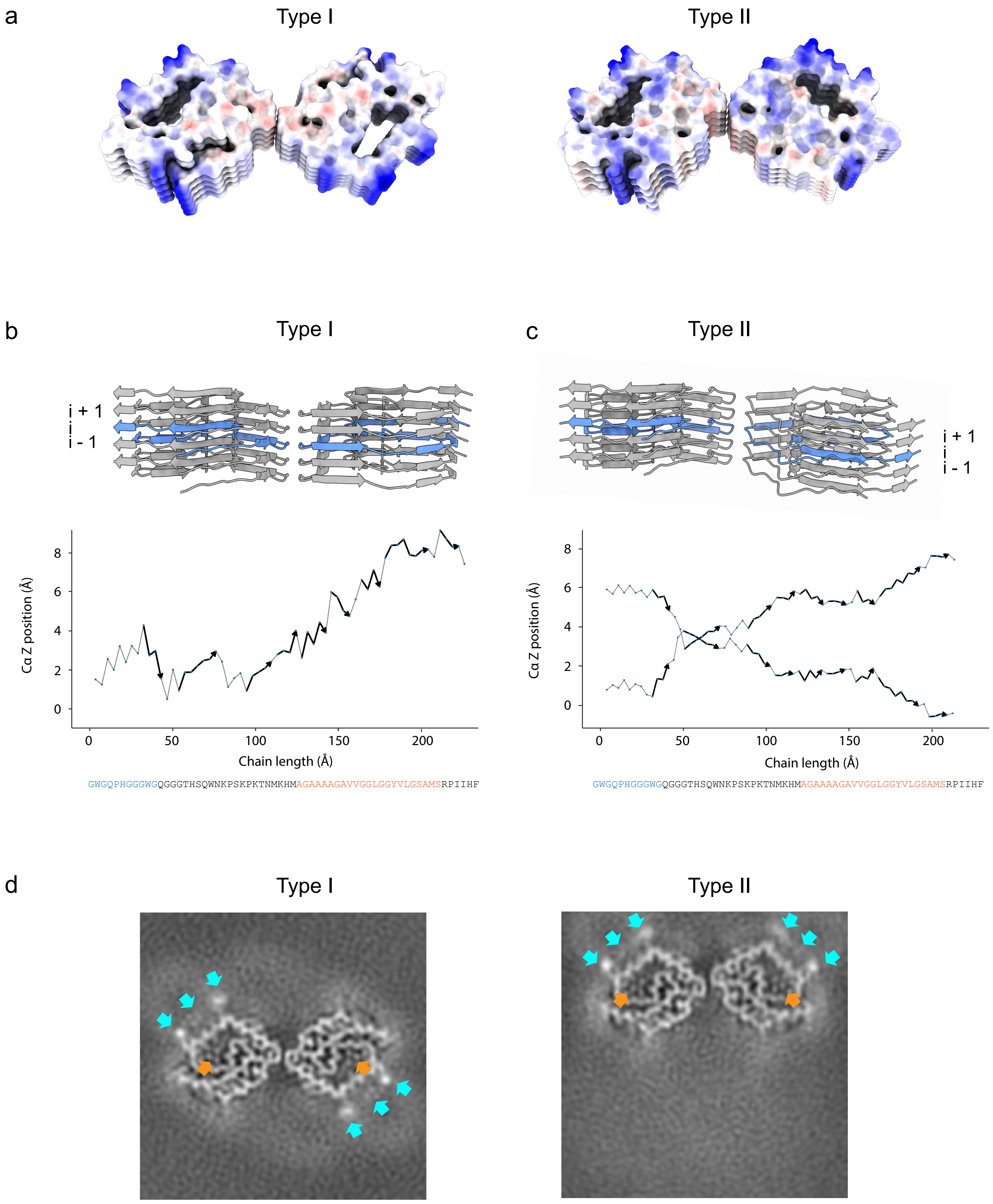
a
Type I
Type II
b
Type I
c
Type II
i + 1
i
i - 1
i + 1
i
i - 1
Cα Z position (Å)
Chain length (Å)
0
2
4
6
8
0
50
100
150
200
GWGQPHGGGWGQGGGTHSQWNKPSKPKTNMKHMAGAAAAGAVVGGLGGYVLGSAMSRPIIHF
GWGQPHGGGWGQGGGTHSQWNKPSKPKTNMKHMAGAAAAGAVVGGLGGYVLGSAMSRPIIHF
d
Type I
Type II

Table S1. Cryo-EM refinement and validation statistics

	**Patient 1 VV-Type 1** (EMDB-26607) (PDB 7UMQ)	**Patient 1 VV-Type 2a**	**Patient 1 VV-Type 2b** (EMDB-26613) (PDB 7UN5)	**Patient 2 MV-Type 1**
**Data collection and processing**				
Voltage (kV)	300	-	-	300
Electron exposure (e-/Å2)	56.5	-	-	57.4
Defocus range (μm)	-2.0 to -0.5	-	-	-2.0 to -0.5
Pixel size (Å)	1.078	-	-	1.054
Symmetry imposed	C2	C1	C1	C2
Movies (no.)	8620	-	-	8330
Initial particles (no.)	440444	-	-	477642
Final particles (no.)	11305	7050	19341	25628
Percent of total particles (%)	2.6	1.6	4.4	5.4
Helical twist (°)	-0.92	-0.89	-0.94	-0.93
Helical rise (Å)	4.82	4.77	4.83	4.73
Map resolution (Å)	3.29	5.50	3.13	3.82
FSC threshold	0.143	0.143	0.143	0.143
**Refinement**				
Initial model used (PDB code)	*De novo*	-	*De novo*	-
Model resolution (Å)<br>At FSC threshold of 0.5	4.37	-	3.98	-
Map sharpening *B* factor ($Å^2$)	102.3	-	108.9	-
Model composition<br>Non-hydrogen atoms<br>Protein residues<br>Ligands	 4380<br>620<br>0	-	 4380<br>620<br>0	-
R.m.s. deviations<br>Bond lengths (Å)<br>Bond angles (°)	 0.023<br>1.9	-	 0.024<br>1.8	-
Validation<br>MolProbity<br>Clashscore<br>Poor rotamers (%)	 1.54<br>2.19<br>0.0	-	 1.31<br>0.93<br>0.0	-
Ramachandran plot<br>Favored (%)<br>Allowed (%)<br>Disallowed (%)	 90.2<br>9.8<br>0.0	-	 90.8<br>9.2<br>0.0	-
